# Rib Hemangiomas: Intriguing Findings from a Systematic Review of Rare Thoracic Tumors

**DOI:** 10.3390/jcm13185586

**Published:** 2024-09-20

**Authors:** Jayant Kumar, Jonathan Magloire, Luis Quintero, Deep Vakil, Himani Bhatt, Noor Kassira, Tamar Levene, Holly Neville

**Affiliations:** 1 Department of General Surgery, Memorial Healthcare System, Pembroke Pines, FL 33028, USA; 2 Department of Surgery and Cancer, Hammersmith Hospital, Imperial College London, London W12 0TS, UK; 3 Division of Pediatric General and Thoracic Surgery, Joe DiMaggio Children’s Hospital, Hollywood, FL 33021, USA

**Keywords:** rib tumors, rib hemangioma, thoracic tumor, hemangioma

## Abstract

**Background:** Bone hemangiomas are rare benign vascular tumors, comprising less than 1% of all bone tumors. They are predominantly found in the vertebral body or skull; rib hemangiomas are particularly rare and are often misdiagnosed as malignant tumors. Given the high malignancy rate of primary rib tumors, understanding rib hemangiomas is crucial to avoid misdiagnosis. **Methods:** A systematic review was conducted according to PRISMA standards. A comprehensive literature search was performed in PubMed, EMBASE, Web of Science, and Scopus. Data on patient demographics, tumor characteristics, and clinical presentation were analyzed using STATA/SE 17. **Results:** From 306 articles, 40 studies including 43 patients met the inclusion criteria. Rib hemangiomas showed a bimodal age distribution, with peaks in patients younger than 30 years (mean age 21.43 ± 5.60 years) and ≥30 years (mean age 59.96 ± 9.70 years). Females were more affected (62.79%) than males (37.21%), with a ratio of 1.69:1. The tumors were most frequently located in mid-thoracic ribs (4–8) and predominantly on the left side of the thorax. The mean tumor size was 7.27 cm, with 76.19% exhibiting osteolytic changes. Clinically, 63.41% of cases were asymptomatic, while symptomatic cases mainly presented with pain. **Conclusions:** Rib hemangiomas, though rare, should be considered in the differential diagnosis of thoracic tumors. They present as well-demarcated lytic lesions with distinct imaging features, and they typically require complete surgical excision, which may be aided with preoperative embolization. Their accurate diagnosis involves a combination of radiologic and clinical evaluation. Further studies are needed to understand the disease’s pathophysiology and to refine diagnostic and treatment protocols.

## 1. Introduction

Bone hemangiomas are benign hamartomatous vascular tumors, accounting for less than 1% of all bone tumors. Approximately 50–80% of these hemangiomas occur in the vertebral body or skull. Vascular lesions affecting the bones present a significant diagnostic challenge and are rarely observed in the thoracic region. Hemangiomas of the rib are particularly rare and are often misdiagnosed as malignant tumors, such as metastatic tumors or primary malignant bone tumors [1,2].

Given that 60–80% of primary rib tumors are malignant, understanding the disease process of rib hemangiomas is crucial. Unlike the hemangiomas commonly found in soft tissues and skin, intra-osseous hemangiomas are relatively rare, with rib localization being particularly uncommon and only sporadic case reports have been documented in the literature [3].

Although bone hemangiomas are benign, they can exhibit local invasiveness and may be mistaken for malignant conditions. Radiologically, differential diagnoses often include metastatic cancers, multiple myeloma, and chondrosarcomas. Rib tumors constitute approximately 5.9% of all primary bone tumors, with a significant majority, about 89%, classified as malignant. Despite their rarity, hemangiomas of the rib should be considered in the differential diagnosis of rib tumors [4].

In this systematic review, we present a comprehensive analysis of the available literature to determine the prevalence, age distribution, location, laterality, and clinical presentation of rib hemangiomas. Our objective is to elucidate the clinical challenges associated with this entity and to develop appropriate diagnostic and therapeutic approaches.

## 2. Methods

### 2.1. Literature Search Methodology

This systematic review was conducted in accordance with the Preferred Reporting Items for Systematic Reviews and Meta-Analyses (PRISMA) guidelines. However, it was not registered in PROSPERO, as this review focuses on a very rare disease that is primarily documented through individual case reports, rather than through large-scale studies or trials that are suitable for meta-analysis. The rarity and specific nature of the condition necessitated a comprehensive review of case reports to synthesize available evidence, which is not typically suited for traditional meta-analytical approaches [5]. A comprehensive literature search was conducted, incorporating articles cataloged within PubMed, EMBASE, Web of Science, and Scopus. The search methodology adhered to the guidelines endorsed by the Cochrane Handbook for Systematic Reviews of Interventions and aligned with the reporting criteria for the meta-analyses of observational studies in epidemiology [6].

The initial search of the databases was completed on 6 May 2024. In this study, a comprehensive search strategy was implemented, combining both controlled terms, such as MeSH or Emtree, and uncontrolled or free terms. The specific terms used were as follows: (“hemangioma” AND “rib neoplasm” OR “rib tumour” OR “rib tumor” AND “thoracic tumour” OR “thoracic tumor”). This combination of terms was conducted following the prescribed protocols of the respective search platforms. Boolean operators (“AND” and “OR”) were employed to refine the search.

The intricacies of the search algorithms are detailed in Supplementary Table S1. A final literature search was performed on 10 June 2024.

This investigation qualified for an exemption from ethical scrutiny because it exclusively employed data from prior publications, and likewise, the requirement for informed consent was waived.

### 2.2. PICOS Question

#### Definition

The systematic review was meticulously structured employing the Patient/Problem, Intervention, Comparison, Outcome, and Study design (PICOS) framework [7]. Our objective is to elucidate the clinical challenges associated with rib hemangiomas and to develop appropriate diagnostic and therapeutic approaches.

The Patient/Problem (P) section focuses on patients with rib hemangiomas—a benign vascular tumor. The specific focus areas include the prevalence, age distribution, location, laterality, and clinical presentation of rib hemangiomas.

The Intervention (I) section includes diagnostic imaging techniques such as chest radiograph, chest computer tomography (CT), and magnetic resonance imaging (MRI), as well as percutaneous needle biopsy in specific cases and therapeutic interventions like complete resection, radiotherapy, trans-arterial embolization, and alcohol injection.

The Comparison (C) section involves comparing rib hemangiomas with other differential diagnoses such as metastatic cancers, primary malignant bone tumors (e.g., chondrosarcoma, osteogenic sarcoma, myeloma, and Ewing sarcoma), and benign bone tumors (e.g., fibrous dysplasia, osteochondroma, aneurysmal bone cyst, and eosinophilic granuloma).

The Outcome (O) section aims for the accurate diagnosis of rib hemangiomas, appropriate therapeutic interventions, and clinical outcomes, including symptom resolution, recurrence rates, and complications.

The Study Design (S) section involves a systematic review of the available literature.

By utilizing the PICOS framework, this systematic review aims to provide a comprehensive understanding of rib hemangiomas, addressing key clinical challenges and facilitating the development of effective diagnostic and therapeutic strategies.

### 2.3. Inclusion and Exclusion Criteria for Study Selection

The previously mentioned searches were completed without restrictions regarding the publication date, type of study, language, or any other delineating parameter. Scholarly articles identified as presumably pertinent within the searched databases were organized and transferred to the Reference Manager. Here, redundant entries and duplicates were removed. The titles and abstracts of the remaining articles were independently assessed by two reviewers—J.K. and J.M.

In case of dispute, a consensus was reached following the arbitration of the chief authors—N.K., T.L., and H.N. Editorials, narrative reviews, and expert opinions were excluded from the analysis. Articles not written in English or those published without any comparative cohort were also excluded.

### 2.4. Outcomes of Interest

The outcomes of interest were age and sex distribution, location, laterality, locations (ribs 1–3, ribs 4–8, or ribs 9–12), osteolytic changes, and symptoms (symptomatic or asymptomatic).

The additional outcomes of interest were diagnostic and treatment approach.

### 2.5. Data Extraction and Analysis

From the eligible studies, a range of variables was systematically harvested. The included attributes were the first author’s name, year of publication, age, sex, location, laterality, size, osteolytic changes, and symptoms.

A data analysis of the qualified studies was executed using STATA/SE 17 (Stata, College Station, TX, USA), and datasets of quantitative and qualitative variables were thoroughly analyzed to estimate the frequency of the respective variables [8,9].

## 3. Results

### 3.1. Characteristics of Included Studies

**Figure 1:**
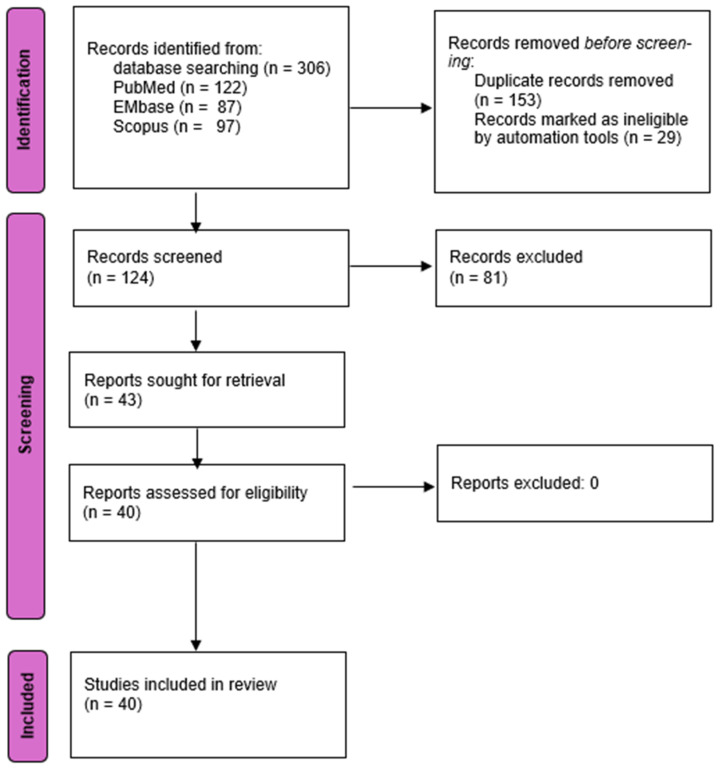
Overview of the search strategy and study selection process following the Preferred Reporting Items for Systematic Reviews and Meta-analyses (PRISMA) protocol in this systematic review (*n*: number of studies).

The preliminary review of the literature yielded 306 articles. Following the elimination of duplicates and a thorough review of titles, abstracts, and full texts, a total of 40 articles including case reports and series were deemed suitable for inclusion. Figure 1 illustrates the PRISMA flowchart that details the results of the search at each stage of the evaluation, consistent with the established study criteria, highlighting the studies earmarked for data extraction. Forty articles comprising 43 patients satisfied the pre-established selection criteria for inclusion (Table 1).

### 3.2. Outcomes of Interest

Rib hemangiomas are a rare entity characterized by the presence of benign vascular tumors within the rib bones. The incidence and clinical presentation of rib hemangiomas exhibit significant variability across different demographic groups, necessitating a thorough understanding of their epidemiology and characteristics. This section delves into various aspects of rib hemangiomas, including age distribution, sex distribution, anatomical location, laterality, tumor size, osteolytic changes, and clinical presentation. Through a detailed examination of these factors, we aim to provide a comprehensive overview that can aid in the effective diagnosis and management of rib hemangiomas (Table 2).

#### 3.2.1. Age Distribution

The incidence of rib hemangioma exhibits a bimodal distribution concerning age-specific mean years. This distribution presents two distinct peaks within the age groups of individuals younger than 30 years and those older than 30 years. Specifically, the mean age for patients 30 years or younger is 21.43 ± 5.60 years, with an observed range from 11 to 29 years. Conversely, for patients older than 30 years, the mean age is significantly higher at 59.96 ± 9.70 years, with an observed range from 44 to 79 years. This bimodal pattern highlights the variation in the prevalence of rib hemangiomas across different age demographics, suggesting that the occurrence of rib hemangiomas is more common in these two distinct age groups. This information underscores the importance of considering age-specific factors in the diagnosis and management of rib hemangiomas (Table 2).

#### 3.2.2. Sex Distribution

Rib hemangioma predominantly affects women more than men (Table 2). The data indicate that out of the total cases studied, 27 were female, accounting for 62.79%, while 16 were male, representing 37.21%. This results in a female-to-male ratio of approximately 1.69:1, suggesting a higher prevalence of rib hemangioma among females compared to males. This gender disparity emphasizes the need for gender-specific considerations in the diagnosis and management of rib hemangiomas.

#### 3.2.3. Location

The distribution of rib hemangiomas across different rib locations reveals a specific pattern (Table 2). These tumors occur with the following frequencies: in ribs 1–3, the frequency is 11.63% (5 cases); in ribs 4–8, the frequency is 76.74% (33 cases); and in ribs 9–12, the frequency is 11.63% (5 cases). This distribution indicates a higher prevalence of rib hemangiomas in the mid-thoracic region, specifically within ribs 4–8.

#### 3.2.4. Laterality

The distribution of rib hemangiomas based on the side of the thorax reveals that these tumors occur with greater frequency on the left side compared to the right side (Table 2). Specifically, 57.14% (24 cases) of rib hemangiomas were located on the left side of the thorax, whereas 42.86% (18 cases) were found on the right side.

#### 3.2.5. Tumor Size

Regarding tumor size, the mean tumor size for the entire studied cohort measured 7.27 cm with a standard deviation of 2.72 cm (Table 2). The median tumor size was 7 cm, with a range extending from the smallest observed size of 2.1 cm to the largest at 16 cm. This distribution indicates a substantial variability in tumor sizes among the patients, with the majority of tumors falling within the interquartile range of 5.5 cm to 9 cm. The skewness of 0.98 suggests a moderate right skew in the data, indicating that a larger number of tumors were smaller in size, while a few were significantly larger.

#### 3.2.6. Osteolytic Changes

The presence of osteolytic changes in rib hemangiomas was assessed in the studied cohort (Table 2). The data reveal that osteolytic changes were present in 32 cases, accounting for 76.19% of the total. Conversely, osteolytic changes were absent in 10 cases, representing 23.81% of the total. This indicates that a significant majority of rib hemangiomas exhibit osteolytic changes, which is an important diagnostic feature to consider when evaluating these tumors.

#### 3.2.7. Clinical Presentation

Regarding the clinical presentation of rib hemangiomas, the data indicate that the majority of cases were asymptomatic (Table 2). Specifically, 26 cases (63.41%) were asymptomatic, while 15 cases (36.59%) presented with symptoms. Among the symptomatic cases, 11 presented with pain, 2 with discomfort, 1 with thoracic outlet syndrome, and 1 with compressive paraparesis. This distribution suggests that while rib hemangiomas are often asymptomatic, a significant portion can present with a range of symptoms, predominantly pain. This variability in clinical presentation highlights the importance of considering rib hemangiomas in differential diagnoses for thoracic symptoms, especially in cases involving unexplained pain or other specific syndromic presentations.

## 4. Discussion

The present study provides a more detailed epidemiological, anatomical, and clinical characterization of rib hemangiomas to address specific gaps in the literature regarding differential diagnosis, management strategies, and the risks associated with biopsy and surgery, thereby offering a more comprehensive and practical guide for clinicians. The study showed that rib hemangiomas exhibit a bimodal age distribution, with peaks in individuals younger than 30 years and those older than 30 years. The mean age for patients 30 years or younger is 21.43 ± 5.60 years, while for those older than 30 years, it is 59.96 ± 9.70 years, suggesting a higher prevalence in these distinct age groups. This highlights the importance of considering age-specific factors in the diagnosis and management of rib hemangiomas. Rib hemangiomas are more prevalent in women, accounting for 62.79% of the studied cohort, compared to 37.21% in men, with a female-to-male ratio of approximately 1.69:1, indicating the need for gender-specific considerations. These tumors are most commonly found in the mid-thoracic region (ribs 4–8), comprising 76.74% of cases, and are more frequent on the left side of the thorax (57.14%) compared to the right side (42.86%).

Rib hemangiomas, typically asymptomatic and often discovered incidentally during radiologic studies, represent a diagnostic challenge due to the wide variety of differential diagnoses for rib lesions. The most common non-neoplastic bone tumor of the thorax is fibrous dysplasia, which, along with other entities such as eosinophilic granuloma, giant cell tumor, and aneurysmal bone cyst, necessitates careful radiologic and clinical evaluation. Fibrous dysplasia presents as a painless, expanding, lytic area in ribs. Solitary plasmacytoma, a rare tumor often associated with latent systemic disease, and multiple myeloma typically present as well-defined, punched-out lytic lesions with associated extra-pleural soft-tissue masses, resembling most metastatic lesions. Aneurysmal bone cysts are expansile lesions with well-defined inner margins on radiographs, and CT scans are particularly useful for delineating both intra-osseous and extra-osseous components of the tumor [12,13,14].

The clinical presentation of rib hemangiomas varies, with 63.41% of cases being asymptomatic and 36.59% presenting with symptoms, predominantly pain. This variability underscores the importance of including rib hemangiomas in differential diagnoses for thoracic symptoms, particularly unexplained pain or specific syndromic presentations. Large lesions can cause pain and swelling, and rarely, hemangiomas arising from the first rib may cause thoracic outlet syndrome, while those close to thoracic vertebrae can present with paresthesia or paresis due to nerve root compression [22,39]. Radiographically, rib hemangiomas generally present as expansile, well-demarcated lytic lesions with intact bone cortex and fine trabeculae. CT or MRI can clearly identify the size and extent of cortical destruction caused by the tumor. Hemangiomas may have a characteristic sunburst-like, honeycombing, or soap bubble appearance and present as well-defined lytic lesions with a coarsened trabecular pattern on imaging. MRI variations depend on the proportion of fat and vascular channels, with T1 hyperintensity due to fat-containing hemangiomas and T2 hyperintensity with flow voids due to vascular channels.

**Figure 2:**
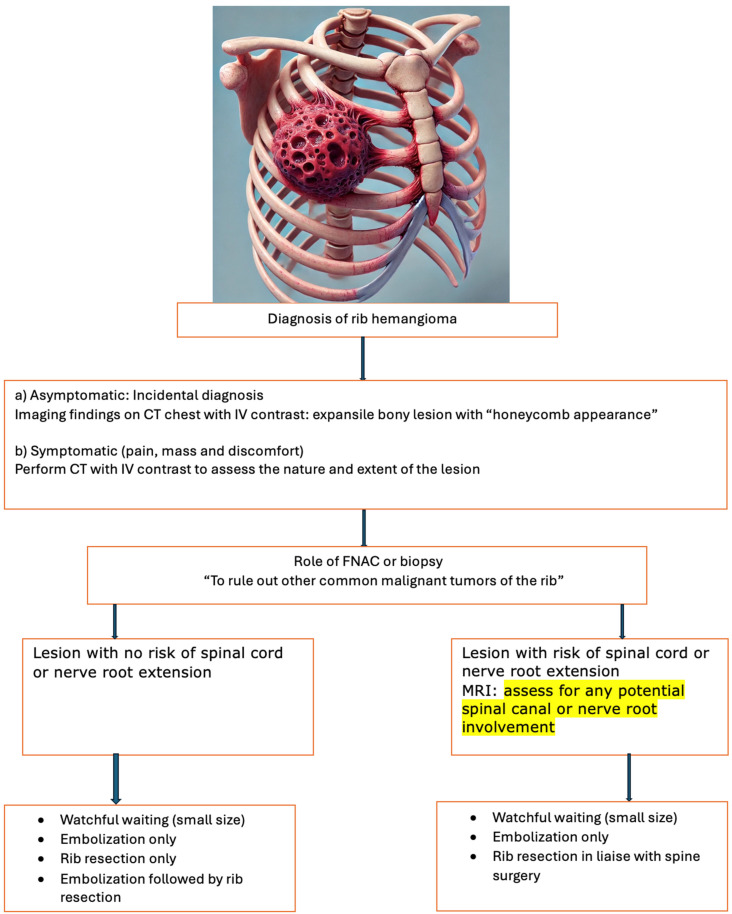
Comprehensive management algorithm for rib hemangioma. Abbreviations: CT—computer tomography; IV—intravenous; FNAC—fine-needle aspiration cytology.

Bone scintigraphy using technetium-99m labeling has shown accumulation in rib tumors, while 18F-FDG PET is considered more useful because it can indicate low or high accumulation. Malignant lesions tend to be 18F-FDG avid, whereas benign lesions generally show lower 18F-FDG avidity. One study reported that the mean SUVmax value in benign rib lesions was 2.5 ± 1.1 [1]. The necessity of preoperative biopsy is controversial due to the risk of seeding or bleeding, advocating for one-step resection surgery instead. Hemangiomas can be classified histologically as cavernous, capillary, venous, or mixed, with cavernous hemangioma being the most common type in peripheral bones. The standard treatment of rib hemangiomas is complete excision with clean surgical margins. Surgical resection was performed in 96.1% of previous cases, with some cases undergoing preoperative embolization to reduce intraoperative blood loss. Resection of the tumors was often performed without biopsy, as the risk of significant bleeding during biopsy is high. The preoperative embolization of large rib hemangiomas can reduce their size, and cement or alcohol injection has been used. Further, in cases where these lesions were close to vertebrae where patients presented with compressive symptoms, repeated embolization was used and the therapy was carried out with significant success. These interventions have shown excellent results in managing symptoms and preventing complications (Figure 2) [43,44,45,46].

Despite its benign nature and indolent progression, hemangioma requires careful differentiation from malignant rib tumors due to the implications for patient management. Given that hemangioma is a benign tumor, it generally does not warrant surgical resection. Consequently, close observation without surgical intervention may be a prudent approach if the tumor is small and is accurately diagnosed as a hemangioma based on imaging. However, biopsy procedures intended to confirm the diagnosis should be approached with caution, as they carry a significant risk of bleeding due to the vascular oknature of the tumor. By addressing these gaps, this study makes significant contributions to the field, particularly in the areas of epidemiology, differential diagnosis, and the management of rib hemangiomas.

The study’s limitations stem from its rarity and reliance on case reports, coupled with its retrospective nature, which collectively constrain the generalizability of its findings. The absence of long-term follow-up and standardized imaging protocols further impedes a comprehensive understanding of the natural history and diagnostic accuracy of rib hemangiomas. Moreover, the study’s failure to investigate genetic or molecular factors limits the depth of insight into the pathogenesis and potential therapeutic approaches for these tumors. To address these gaps, future research should prioritize a prospective, multi-center approach, integrating larger sample sizes, standardized imaging criteria, and the inclusion of longitudinal data alongside molecular analyses. Such methodological advancements would significantly enhance the robustness and applicability of the findings, ultimately contributing to a more precise and nuanced understanding of rib hemangiomas.

## 5. Conclusions

To recapitulate, rib hemangiomas are often incidental findings with significant variability in presentation and demographic distribution. Accurate diagnosis relies on a combination of radiologic imaging and clinical evaluation, and the primary treatment remains surgical excision, often supplemented by preoperative interventions to manage symptoms and prevent complications. Further studies are essential to deepen the understanding of the disease pathophysiology of rib hemangiomas and to refine diagnostic and treatment protocols.

## Figures and Tables

**Table 1:** Summary of included articles highlighting pertinent attributes.

Author	Age (Years)	Sex	Rib Number (s) or Location	Laterality	Osteolytic Changes	Size (cm)	Presentation
Abrao [2]	48	F	7	L	P	5 × 3.8	AS
Hashimoto [10]	64	F	5	L	P	8 × 4 × 2.5	AS
Jia [11]	54	F	9	R	P	7.5 × 5.2 × 4.5	AS
Tew [12]	20	M	5	R	P	5	AS
Jain [13]	26	F	8	R	P	6.5 × 3 × 4.5	Pain
Bouchikh [14]	17	M	6	R	P	10	AS
Burke [15]	16	M	8	L	NP	7.2 × 3.7 × 4.0	Pain
Lmai [16]	57	F	1	R	P	6	AS
Yamamoto [17]	73	F	8	L	P	12 × 11 × 11	AS
Memduh [18]	30	M	8	R	NP	6 × 4	NR
Alloubi [19]	63	F	3	L	P	4.5 × 6	AS
Mishra [20]	25	F	2	L	P	7 × 9 × 5	Pain
Haro [21]	79	F	6	L	P	2.9 × 2.5 × 1.9	AS
Shaik [22]	21	M	5	L	P	NR	NR
Desmukh [23]	18	F	3	R	P	NR	AS
Weinardt [24]	76	F	4, 5, 6	L	P	10	Back Pain
Park [25]	63	F	6	L	P	5.5 × 2.5 × 1.5	AS
Zhu [26]	47	F	7	R	P	6.5 × 3.5	AS
Puri [27]	63	F	4	L	P	6.5 × 5 × 2.5	Pain
Itabashi [1]	68	M	4	L	P	1.8 × 1.6 × 2.1	AS
Tasuda [28]	58	M	11	R	P	NR	AS
Tasuda [28]	49	M	4	R	NP	NR	AS
Huang [29]	44	F	R	L	P	8.5	AS
Morkan [30]	23	M	multiple	L	P	5 × 3.8 × 2.5	Pain
Liu [31]	27	F	10	R	P	8.2 × 8 × 7.1	AS
Feldman [32]	53	F	7	R	NP	NR	Discomfort
Ortega [33]	11	F	6	L	NP	NR	Pain
Ortega [33]	14	M	multiple	NR	P	NR	Pain
Ortega [33]	65	F	8	L	NP	NR	Pain
Kuo [3]	56	F	7	L	P	16 × 7 × 6	AS
Filosso [34]	61	M	12	L	NP	5	Pain
Clements [35]	76	M	3	R	NP	7.5 × 3	AS
Ogose [36]	59	F	4	R	NA	NR	AS
Okumura [37]	59	F	7	L	P	3.5 × 2.5 × 1.5	AS
Shimizu [38]	59	M	4	L	P	7.5	AS
Yeow [39]	50	F	1	R	NP	NR	Discomfort
Roy [40]	45	M	7	R	P	NR	Pain
Ovali [41]	25	F	8	L	P	4 × 4 × 7	AS
Nakamura [42]	74	M	5	L	P	9.5 × 6.5 × 3.0	Pain
Sirmali [43]	28	F	5	R	P	7 × 9 × 5	Pain
Ceberut [44]	62	M	5	R	P	6 × 5 × 3.5	AS
Cakir [45]	54	F	8	L	P	9 × 6 × 5	AS
Gourgiotis [46]	29	F	7	L	NP	4.5	AS

Abbreviations—AS: asymptomatic; F: female; L: left; M: male; NP: not present; NR: not reported; P: present; R: right.

**Table 2:** Comprehensive summary of patient demographics and characteristics from included articles.

Category	Subcategory	Observations	Percent (%)	Mean	Std dev	Min	Max
Age	>30 years	28	-	59.96	9.70	44	79
	≤30 years	14	-	21.43	5.60	11	29
Sex distribution	Female	27	62.79	-	-	-	-
	Male	16	37.21	-	-	-	-
Location	A: Ribs 1–3	05	11.63	-	-	-	-
	B: Ribs 4–8	33	76.74	-	-	-	-
	C: Ribs 9–12	05	11.63	-	-	-	-
Laterality	Left	24	57.14	-	-	-	-
	Right	18	42.86	-	-	-	-
Clinical presentation	Asymptomatic	26	63.41	-	-	-	-
	Symptomatic	15	36.59	-	-	-	-
Osteolytic changes	Present	32	76.19	-	-	-	-
	Absent	10	23.81	-	-	-	-

Abbreviations—Max: maximum; Min: minimum; Std dev: standard deviation.

## Data Availability

All data that support the conclusions of this manuscript are included in the Supplementary Materials.

## Supplementary Materials

The following supporting information can be downloaded at: https://www.mdpi.com/article/10.3390/jcm13185586/s1, Table S1: Databases and search strategy.
