# Supplementary material for: Rib Hemangiomas: Intriguing Findings from a Systematic Review of Rare Thoracic Tumors
Source: J Clin Med. 2024 Sep 20;13(18):5586. doi: 10.3390/jcm13185586 (PMC11433624; doi:10.3390/jcm13185586)
Supplement: Supplementary file 1 [file jcm-13-05586-s001.zip › jcm-3103107-supplementary.pdf]

Supplementary Table S1. Databases and search strategy.

	Database	Search Term and strategy
1	PubMed	("Hemangioma"[Mesh] OR "hemangioma") AND ("Rib Neoplasms"[Mesh] OR "rib neoplasm" OR "rib tumour" "rib tumor" OR "rib hemangioma") AND ("thoracic tumour" OR "thoracic tumor")
2	Embase	('hemangioma'/exp OR 'hemangioma') AND ('rib tumor'/exp OR 'rib tumor' OR 'rib neoplasm' OR 'rib tumour' OR ' rib tumour' OR 'rib hemangioma') AND ('thoracic tumour' OR 'thoracic tumor')
3	Web of Science	("hemangioma") AND ("rib neoplasm" OR "rib tumour" OR "rib tumor" OR "rib hemangioma") AND ("thoracic tumour" OR "thoracic tumor")
4	Scopus	(TITLE-ABS-KEY ("Hemangioma") AND TITLE-ABS-KEY ("Rib Neoplasms" OR "rib neoplasm" OR "rib tumour" OR "rib tumor" OR "rib hemangioma") AND TITLE-ABS-KEY ("thoracic tumour" OR "thoracic tumor"))

Abbreviations: MeSH: medical subject headings
